# Hsa-miR-375 is a predictor of local control in early stage breast cancer

**DOI:** 10.1186/s13148-016-0198-1

**Published:** 2016-03-08

**Authors:** Franz Zehentmayr, Cornelia Hauser-Kronberger, Barbara Zellinger, Falk Hlubek, Claudia Schuster, Ulrich Bodenhofer, Gerd Fastner, Heinz Deutschmann, Philipp Steininger, Roland Reitsamer, Thorsten Fischer, Felix Sedlmayer

**Affiliations:** Department of Radiation Oncology, Paracelsus Medical University, SALK, Müllner Hauptstraße 48, A-5020 Salzburg, Austria; radART - Institute for Research and Development on Advanced Radiation Technologies, Paracelsus Medical University, Salzburg, Austria; Department of Pathology, Paracelsus Medical University, SALK, Salzburg, Austria; Department of Gynecology and Obstetrics, Paracelsus Medical University, SALK, Salzburg, Austria; Department of Pathology, Ludwig Maximilian University, Munich, Germany; Institute of Bioinformatics, Johannes Kepler University, Linz, Austria

**Keywords:** MicroRNAs, hsa-miR-375, Early stage breast cancer, Local control, Predictive markers

## Abstract

**Background:**

A long-term analysis by the Early Breast Cancer Trialist Group (EBCTG) revealed a strong correlation between local control and cancer-specific mortality. MicroRNAs (miRs), short (20–25 nucleotides) non-coding RNAs, have been described as prognosticators and predictors for breast cancer in recent years. The aim of the current study was to identify miRs that can predict local control after breast conserving therapy (BCT) in early stage breast cancer.

**Results:**

Clinical data of 46 early stage breast cancer patients with local relapse after BCT were selected from the institutional database. These patients were matched to 101 control patients showing identical clinical features but without local relapse. The study was conducted in two steps. (1) In the pilot study, 32 patients (16 relapses versus 16 controls) were screened for the most de-regulated microRNAs (= candidate microRNAs) in a panel of 1250 miRs by microarray technology. Eight miRs were found to be significantly de-regulated. (2) In the validation study, the candidate microRNAs were analyzed in an independent cohort of 115 patients (30 relapses versus 85 controls) with reverse transcription quantitative polymerase chain reaction (RT-qPCR). From these eight candidates, hsa-miR-375 could be validated. Its median fold change was 2.28 (Mann-Whitney *U* test, corrected *p* value = 0.008). In the log-rank analysis, high expression levels of hsa-miR-375 correlated with a significantly higher risk of local relapse (*p* = 0.003). In a multivariate analysis (forward stepwise regression) including established predictors and prognosticators, hsa-miR-375 was the only variable that was able to distinguish the statistical significance between relapse and control groups (raw *p* value = 0.000195 HR = 0.76, 95 % CI 0.66–0.88; corrected *p* value = 0.005).

**Conclusions:**

Hsa-miR-375 predicts local control in patient with early stage breast cancer, especially in estrogen receptor α (ER-α)-positive patients. It can therefore serve as an additional molecular marker for treatment choice independently from known predictors and prognosticators. Validation in larger prospective studies is warranted.

**Electronic supplementary material:**

The online version of this article (doi:10.1186/s13148-016-0198-1) contains supplementary material, which is available to authorized users.

## Background

In 2014, the estimated number of deaths from breast cancer in Europe is 89,300 [1] and 40,000 in the US [2]. Every uncontrolled primary tumor comprises the risk of subsequent metastatic disease, and the younger the patient, the more likely she will die from it. A long-term analysis by the Early Breast Cancer Trialist Group (EBCTG) showed a 4:1 relation: if four local relapses can be avoided at year 10, one breast cancer-specific death can be avoided at year 15 [3, 4]. Due to advances in all disciplines related to breast conserving therapy (BCT), the local relapse rate is generally low, with age still appearing as the most important prognosticator and predictor for local relapse. Nowadays, the estimates for age adjusted annual in-breast recurrence rates are 0.4–0.7 % for patients >50 years, 0.72–1.2 % in the age group 41–50 years, and 0.72–2 % for patients younger than 40 years, respectively [5–7].

Additionally, many other patient- and therapy-related factors as well as molecular markers have been described to influence clinical outcome [8–10]. Seventy percent of invasive breast cancers show increased expression of the estrogen receptor α (ER-α). Up-regulation of ER-α is an early event in tumorigenesis and cancer progression [11, 12]. In recent years, microRNAs (miRs) were described as potential prognosticators and predictors for breast cancer. MiRs are short non-coding RNAs (21–25 nucleotides), which play a role in human cancer development both as oncogenes and tumor suppressors. More than 50 % of the known miR genes are located in genetically altered regions of the genome [13]. In 2005, Iorio et al. suggested a miR signature for breast cancer. This panel of de-regulated miRs correlated with known clinical and biological features such as hormonal receptor status, tumor size, lymph node status, vascular invasion, proliferation index, and p53 [14]. Recently, scientific interest has partially focused on the connection between miRs and hormone receptors [15–20]. About 15 miRs have been identified as regulators of ER-α [21].

Thus far, only one clinical study investigated the role of miRs with respect to local control (LC) in early stage breast cancer [22]. Zhou et al. showed that hsa-miR-9 could discriminate between patients with and without local relapse. Interestingly, the effect was most pronounced in ER-α positive patients. In order to further elucidate the importance of miRs for LC in early stage breast cancer, the aim of the current study was to identify miRs that can differentiate between patients with and without local relapse after BCT. This is especially important in the context of individualized local treatment selection.

## Results

### Patients

Potentially prognostic characteristics were distributed without significant differences between relapse and control groups (Table 1). The median follow-up in the pilot cohort was 126 months (range 26–200 months), and in the validation cohort, it was 136 months (range 17–207 months). The median time to local relapse was 37 months (range 15–123 months) in the pilot cohort and 74 months (range 35–185 months) in the validation cohort (Table 2).

**Table 1:** Patient and treatment characteristics

Patient and treatment characteristics							
Parameters		Pilot phase *n* = 32			Validation phase *n* = 115		
		Relapse *n* = 16	Control *n* = 16	*p* value	Relapse *n* = 30	Control *n* = 85	*p* value
Age at diagnosis (years)	Median	53.5	52	0.88	52	54	0.59
	Range	36–71	35–74		33–79	37–78	
Menopause (*n*)	No	4 (25 %)	4 (25 %)	1.0	11 (36.7 %)	32 (37.6 %)	0.37
	Yes	8 (50 %)	8 (50 %)		17 (56.7 %)	40 (47.1 %)	
	Unclear	4 (25 %)	4 (25 %)		2 (6.7 %)	13 (15.3 %)	
T (*n*)	T1	10 (62.5 %)	10 (62.5 %)	1.0	26 (86.7 %)	75 (88.2 %)	0.82
	T2	6 (37.5 %)	6 (37.5 %)		4 (13.3 %)	10 (11.8 %)	
N (*n*)	N0	13 (81.3 %)	13 (81.3 %)	1.0	24 (80 %)	67 (78.8 %)	0.88
	N1	2 (12.5 %)	2 (12.5 %)		6 (20 %)	18 (21.1 %)	
	N2	1 (6.3 %)	1 (6.3 %)		0	0	
M (*n*)	M0	16 (100 %)	16 (100 %)	1.0	30 (100 %)	85 (100 %)	1.0
	M1	0	0		0	0	
Grading (*n*)	G1	1 (6.3 %)	0	0.87	3 (10 %)	8 (9.4 %)	0.75
	G2	8 (50 %)	9 (56.3 %)		18 (60 %)	55 (64.7 %)	
	G3	7 (43.8 %)	7 (43.8 %)		9 (30 %)	22 (25.9 %)	
Histology (*n*)	IDC	13 (81.3 %)	15 (93.8 %)	0.56	22 (73.3 %)	63 (74.1 %)	0.98
	ILC	3 (18.8 %)	1 (6.3 %)		5 (16.7 %)	12 (14.1 %)	
	Tubular	0	0		2 (6.7 %)	6 (7.1 %)	
	Other	0	0		1 (3.3 %)	4 (4.7 %)	
In situ component (*n*)	Yes	14 (87.5 %)	10 (62.5 %)	0.24	17 (56.7 %)	47 (55.3 %)	0.97
	No	2 (12.5 %)	6 (6.25 %)		13 (43.3 %)	35 (41.2 %)	
	Lymphangiosis	0	0		0	1 (1.2 %)	
	Not stated	0	0		0	2 (2.4 %)	
Receptors (*n*)	ER positive	8 (50 %)	10 (62.5 %)	0.56	23 (76 %)	67 (78.8 %)	0.81
	ER negative	8 (50 %)	6 (37.5 %)		7 (23.3 %)	18 (21.2 %)	
	PR positive	8 (50 %)	8 (50 %)	0.56	19 (63.3 %)	62 (72.9 %)	0.32
	PR negative	8 (50 %)	8 (50 %)		11 (36.7 %)	23 (27.1 %)	
her2neu	Positive	8 (50 %)	4 (25 %)	0.24	11 (37 %)	24 (28 %)	0.81
	Negative	5 (31 %)	9 (56 %)		18 (60 %)	44 (52 %)	
	Not assessable	3 (19 %)	3 (19 %)		1 (3 %)	17 (20 %)	
Proliferation index	ki67 < 20 %	10 (63 %)	6 (38 %)	0.24	15 (50 %)	49 (58 %)	0.78
	ki67 > 20 %	5 (31 %)	9 (56 %)		10 (33 %)	26 (31 %)	
	Not assessable	1 (6 %)	1 (6 %)		5 (17 %)	10 (11 %)	
Boost (*n*)	Intraoperative	8 (50 %)	8 (50 %)	1.0	11 (36.7 %)	34 (40 %)	0.91
	Percutaneous	8 (50 %)	8 (50 %)		16 (53.3 %)	51 (60 %)	
	None	0	0		3 (10 %)	0	
Boost dose (Gy)	Dose intraoperative	10 Gy	10 Gy	0.84	10 Gy	10 Gy	0.58
	Dose percutaneous	12 Gy	12 Gy		12 (15–19) Gy	12 (9–19) Gy	
WBRT dose (Gy)	Median	54 Gy	54 Gy	0.78	54 Gy	54 Gy	0.68
	Range	51.2–61.2 Gy	51.2–57.8 Gy		51–63 Gy	51–57.6 Gy	
Surgery (*n*)	BCT	16 (100 %)	15 (93.8 %)	0.78	30 (100 %)	85 (100 %)	1.0
	Mastectomy	0	1 (6.3 %)		0	0	
Re-Excisition (*n*)	Yes	8 (50 %)	4 (25 %)	0.24	12 (40 %)	33 (38.8 %)	0.91
	No	8 (50 %)	12 (75 %)		18 (60 %)	52 (61.2 %)	
Year of surgery (*n*)	Before 1998	5 (31.3 %)	5 (31.3 %)	1.0	14 (46.7 %)	38 (44.7 %)	0.85
	Since 1998	11 (68.8 %)	11 (68.8 %)		16 (53.3 %)	47 (55.3 %)	
Chemotherapy (*n*)	Yes	8 (50 %)	6 (37.5 %)	0.56	11 (36.7 %)	20 (23.5 %)	0.18
	No	8 (50 %)	10 (62.5 %)		19 (63.3 %)	65 (76.5 %)	
Hormonal treatment (*n*)	Yes	8 (50 %)	7 (43.8 %)	0.78	16 (53.3 %)	56 (65.9 %)	0.27
	No	8 (50 %)	9 (56.3 %)		13 (43.3 %)	26 (30.6 %)	
	Unclear	0	0		1 (3.3 %)	3 (3.5 %)	
Tumor burden in biopsy (%)	Median	70	50	0.34	70	50	0.52
	Range	10–90	10–90		10–90	10–90	

Patient and treatment characteristics as well as the relative tumor burden in the samples are shown. The relapse group and control were compared with the Mann-Whitney *U* test. Neither in the pilot nor in the validation phase statistically significant differences of potentially prognostic parameters were detected between the relapse and the control group. The stainings for her2 and ki-67 were performed according to the standard procedures implemented at the Department of Pathology. Because some of the specimens were quite old (minimum 5 years) in some cases, the stainings—even on repetition—did not yield valid result due to technical problems with the non-adhesive FFPE sections

**Table 2:** Follow-up and clinical outcome

Clinical outcome					
Parameters		Pilot phase *n* = 32		Validation phase *n* = 115	
		Relapse *n* = 16	Control *n* = 16	Relapse *n* = 30	Control *n* = 85
Time to local relapse (months)	Median	37	x	74	x
	Range	15–123	x	35–185	x
Time to distant metastasis (months)	Median	61	x	70	49.5
	Range	20–96	x	27–118	9–109
Follow-up (months)	Median	121.5	130.5	125	140
	Range	26–192	72–200	44–214	17–207
	Lost to follow-up (*n*)	0	0	0	1 (1.2 %)
Cancer specific deaths (*n*)		4 (25 %)	0	7 (23.3 %)	2 (2.4 %)

Summary follow-up and clinical outcome in the pilot and validation phases

### Pilot phase

**Fig. 1:**
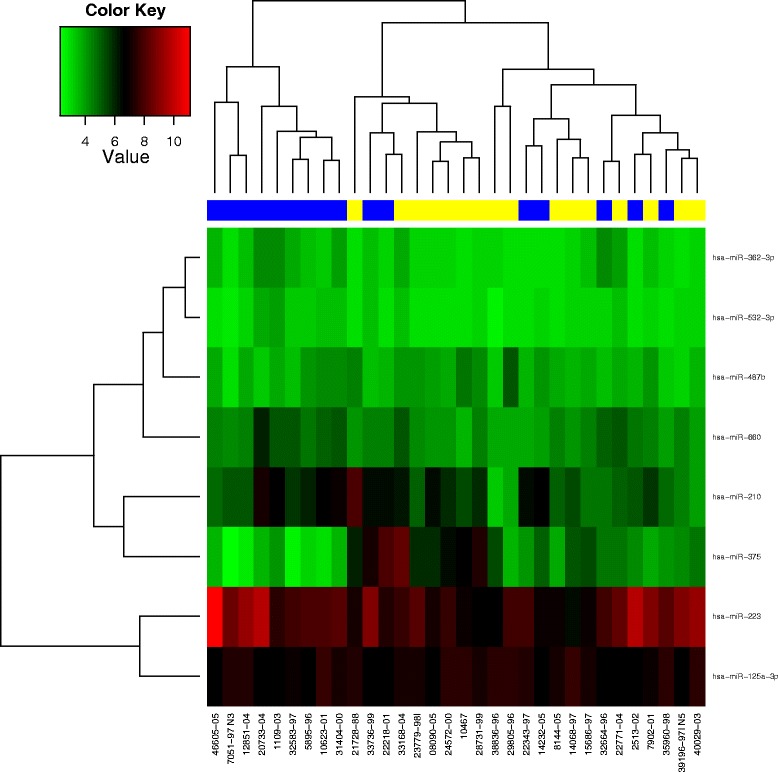
By means of hierarchical clustering, a heat-map was generated. The dendrogram on *top* depicts the grouping of patients according to their pattern of candidate miRs (*yellow*: patient with local relapse; *blue*: patient without local relapse). The intensity values of a given miR are shown in *green* (low intensity) and *red* (high intensity). On the *right side*, the eight candidate miRs are listed. At the *bottom*, the sample numbers are shown. The first knot in the dendrogram separates a group of patients without relapse (pattern B) from the rest of the cohort (pattern A)

**Fig. 2:**
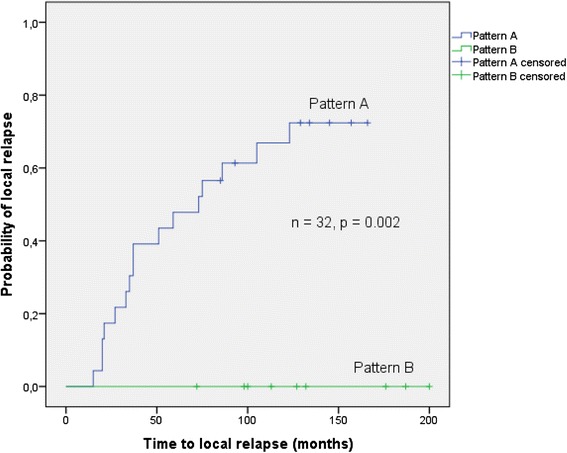
This plot shows the log-rank comparison of *pattern A* versus *pattern B*. The pattern of eight miRs (hsa-miR-362-3p, hsa-miR-532-3p, hsa-miR-487b, hsa-miR-660, hsa-miR-210, hsa-miR-375, hsa-miR-223, hsa-miR-125a-3p) was able to differentiate between relapse group and control group

In the pilot study, five miRs showed a significant fold change between relapse patients and their controls (linear models for microarray analysis (LIMMA), raw *p* value <0.05): hsa-miR-362-3p, hsa-miR-660, hsa-miR-375, hsa-miR-223, and hsa-miR-125a-3p. Three miRs had a fold change (relapse versus control) that was close to significance (LIMMA, raw *p* value = 0.05): hsa-miR-532-3p, hsa-miR-487b, and hsa-miR-210. Hierarchical clustering of these eight miRs revealed two main groups with different miR-expression patterns (Fig. 1). One group only consisted of controls (pattern B), while relapse patients formed the majority of the other group (pattern A). The log-rank comparison revealed a significant *p* value of 0.002 (Fig. 2). The levels of hsa-miR-375 were significantly higher in the relapse group, with a raw *p* value of 0.009 (LIMMA) and a fold change of 2.97 between relapse and controls, which is illustrated in (Additional file 1: Figure S2). The microarray data were deposited in NCBI Gene Expression Omnibus under the accession number GSE69951; they can be accessed via the following link: http://www.ncbi.nlm.nih.gov/geo/query/acc.cgi?acc=GSE69951.

### Validation phase

**Fig. 3:**
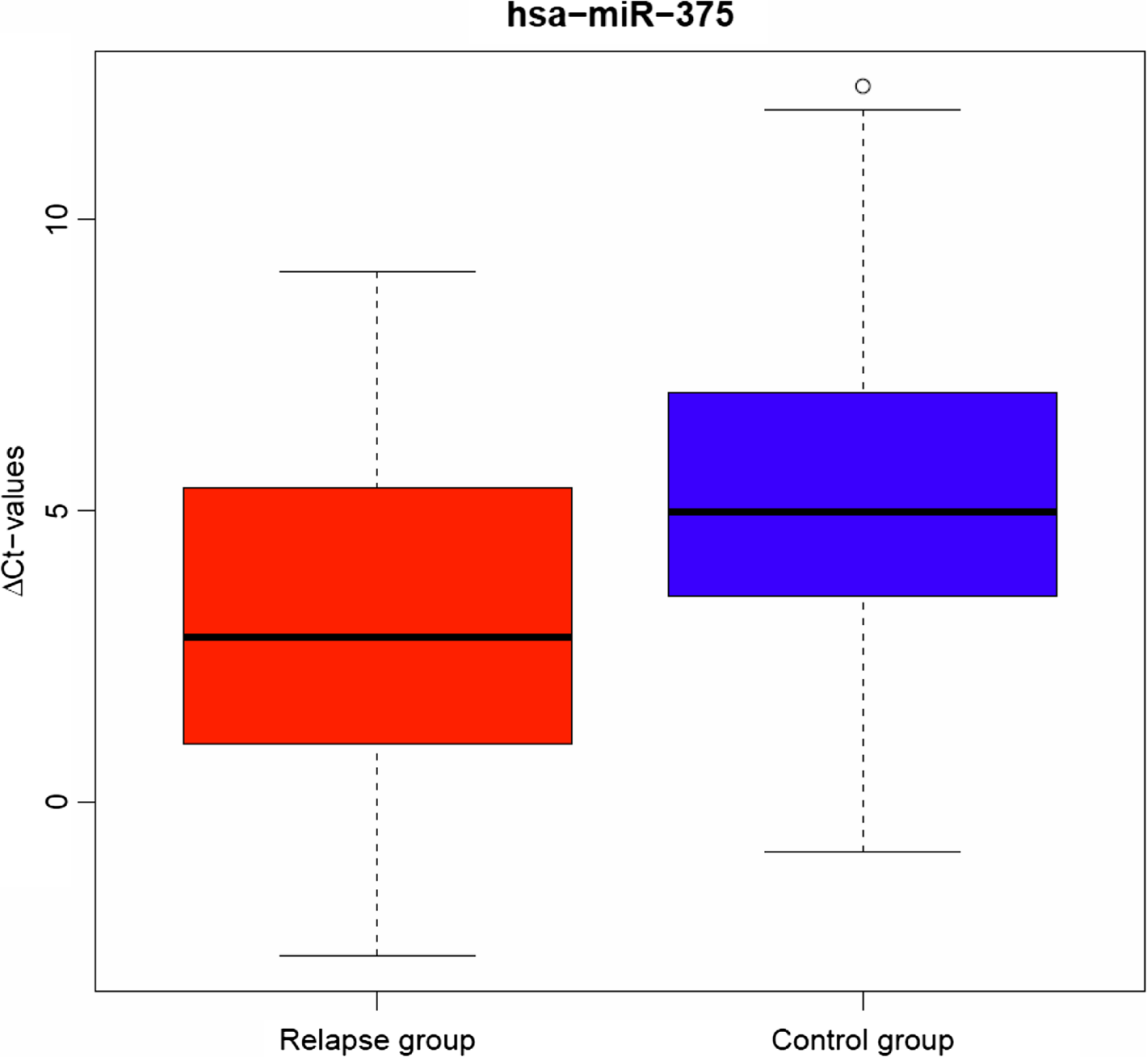
Hsa-miR-375 was the most prominent miR to differentiate between relapse and control group. The *y-axis* shows the ∆Ct values of hsa-miR-375 in relapse and control group; therefore, a high ∆Ct value means low expression of hsa-miR-375

**Fig. 4:**
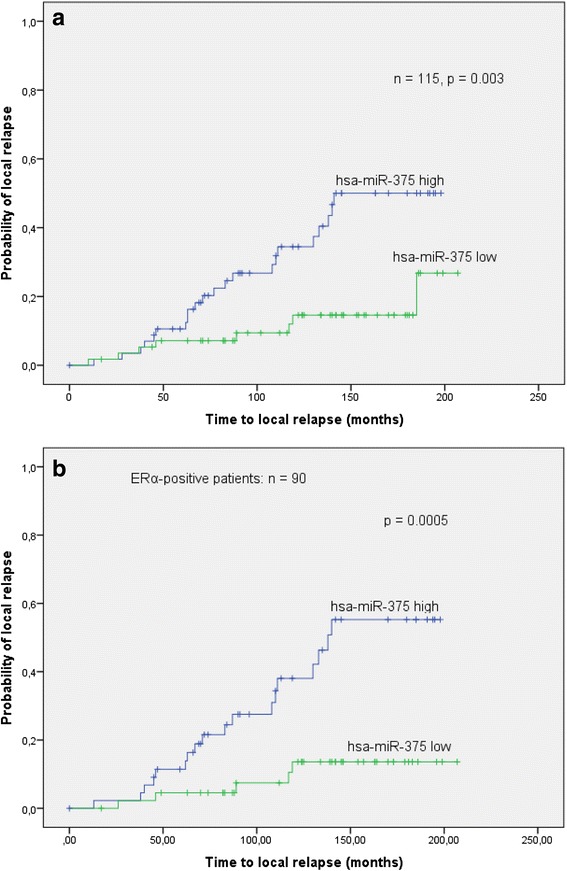
**a** In a time-to-event analysis (event = local relapse), hsa-miR-375 was able to separate the relapse from the control group (log-rank *p* = 0.003): high expression levels were correlated with a higher probability of local relapse. **b** In a subgroup analysis of only *ERα*-positive patients, the differentiation between relapse and control based on the levels of hsa-miR-375 was more pronounced (log-rank *p* = 0.0005)

For further validation, we selected the eight miRs that were able to differentiate between relapse and controls in the hierarchical cluster analysis. Of these eight candidate miRs, hsa-miR-375 could be validated by reverse transcription quantitative polymerase chain reaction (RT-qPCR), in the sense that it was able to differentiate between relapse and control group in an independent patient cohort (Table 3). The levels of hsa-miR-375 were—similar to the pilot phase—significantly higher in the relapse group compared to controls (Mann-Whitney *U* test, raw *p* value = 0.001; *p* value corrected according to Bonferroni = 0.008) with a median fold change of 2.28 (Table 3). The box plot in Fig. 3 shows the elevated levels of hsa-miR-375 in relapse patients compared to controls (Fig. 3). In the log-rank analysis (event = local relapse), high expression levels of hsa-miR-375 correlated to a significantly increased risk of local relapse (*p* value = 0.003; Fig. 4a). When tested in the multivariate analysis (forward stepwise regression) together with the patient- and treatment-related parameters listed in Table 1, hsa-miR-375 was the only variable that was able to distinguish the statistical significance between the relapse and control groups (raw *p* value = 0.000195 HR = 0.76, 95 % CI 0.66–0.88; *p* value corrected according to Bonferroni = 0.005).

**Table 3:** Summary and comparison of fold change, ∆Ct values, and PCR efficiency

	Pilot phase	Validation phase					
miRNA	Median fold change relapse/control	Relapse (∆Ct − median)	Control (∆Ct − median)	Median fold change relapse/control	PCR efficiency	Raw *p* value	Corrected *p* value
hsa-miR-660	0.77	5.13	7.31	4.53	2.00	<0.001	<0.001
hsa-miR-375	2.97	3.24	4.43	2.28	1.89	0.001	0.008
hsa-miR-125a-3p	1.26	6.79	6.91	1.09	2.02	0.448	1.000
hsa-miR-362-3p	0.80	7.25	7.82	1.48	1.98	0.093	0.744
hsa-miR-210	0.51	6.15	5.21	0.52	1.95	0.121	1.000
hsa-miR-223	0.58	2.81	3.13	1.25	1.98	0.806	1.000
hsa-miR-487b	1.22	6.93	7.30	1.29	2.10	0.373	1.000
hsa-miR-532-3p	0.90	6.22	6.30	1.05	2.15	0.679	1.000

Eight candidate miRs were selected in the pilot phase and further analyzed in an independent cohort by RT-qPCR. Both in the pilot and the validation cohort, the levels of hsa-miR-375 were significantly higher in the relapse group (raw *p* value = 0.001, corrected *p* value = 0.008). For calculation of the relative miR expression, the Ct values of the reference gene were subtracted from the Ct values of the target miR. The fold change was estimated with the *∆∆*Ct method as described by Livak. Correlations were tested for statistical significance with the non-parametric Mann-Whitney test, *p* values were corrected for multiple testing according to Bonferroni

In the subgroup of ER-α-positive patients, the difference in hsa-miR-375 levels between the relapse and control patients was also significant (*p* value = 0.001, Mann-Whitney *U* test), whereas in ER-α-negative patients, it was not (*p* = 0.495, Mann-Whitney *U* test). The log-rank analysis (event = local relapse) showed that high expression levels of hsa-miR-375 correlated to a higher probability of local relapse, which was more significant than in the whole patient cohort (*p* = 0.0005, see Fig. 4b).

The comparison of the hsa-miR-375 levels in ER-α-positive to triple negative patients showed a fold change of 4.17 (*p* value = 0.24, Mann-Whitney *U* test). Further subgroup comparisons showed that the levels of hsa-miR-375 were higher in the ER-α-positive patients compared to those in the ER-α-negative patients but without significance (*p* value 0.53). The same applies to her2-negative patients compared with her2-positive patients 1.52 (*p* value 0.66). For further details, please see Additional file 2: Table S2.

### External validation

Hsa-miR-375 was cross-validated in randomly selected samples of five relapses and their 14 corresponding controls from the validation cohort. The result of the first validation step concerning hsa-miR-375 could be verified with a median fold change of 2.84 and a *p* value of 0.019 (Mann-Whitney *U* test).

## Discussion

Although the clinical outcome in early stage breast cancer patients is generally good, some patients experience local relapse after BCT. Apart from clinical features, biomarkers can add to the optimal treatment choice. In the current study, we were able to identify and validate hsa-miR-375 as a potential predictor for LC after BCT. Our results allow us to assume that patients with high levels of hsa-miR-375 in the primary tumor have an increased risk of local relapse. Therefore, hsa-miR-375 is a new potential predictor for LC in early stage breast cancer independent of the known clinical and molecular markers. It could also become an additional selection tool for the choice of primary operative treatment. Patients with a high level of hsa-miR-375 show a higher risk of local relapse; however, threshold values have yet to be defined before clinical translation. These patients might be possible candidates for more aggressive local treatment, i.e., mastectomy or dose-intensified adjuvant radiotherapy following breast conserving surgery*.*

The first study to show that miRs in general play a role in breast cancer was published by Iorio and colleagues. The authors compared miR-expression levels by microarray technology in 76 primary tumors versus 34 normal tissues and identified a set of 15 miRs that could differentiate between tumor and normal tissue with 100 % accuracy [14].

Recent studies have focused on the relationship between ER-α and miR-expression levels. This is of importance for the current study since 80 % of the 147 patients we analyzed were ER-α positive. Profiling studies identified numerous miRs that are associated with either ER-α-positive or ER-α-negative tumors [14, 23–26]. The web of miRs involved in the regulation of the estrogen receptor activity is complex*.* One of these is hsa-miR-375, which plays a role in various types of cancer such as prostate [27], stomach [28], lung [29], and pancreas [30]. A connection between hsa-miR-375 and ER-α positivity was described in vitro [11, 31], in an interventional study in rodents [12] as well as in the clinical context of Tamoxifen resistance [31–34] and progression from normal tissue through lobular carcinoma in situ (LCIS) to invasive lobular carcinoma (ILC) [35].

Souza et al. compared the profiles of miRs and mRNA in cell culture. Inhibition of hsa-miR-375 reduced ER-α activation and cell proliferation. Dexamethasone-induced RAS-related protein 1 (RASD1) was found to be a direct target of hsa-miR-375, which negatively regulates ER-α expression. Hsa-miR-375 was up-regulated in ER-α-positive cells compared to ER-α-negative cells and normal cells. This led to the assumption that up-regulation of hsa-miR-375 is both a key driver of proliferation and an early event in tumorigenesis in ER-α positive tissues [34].

In an interventional study in rodents, Munagala et al. could verify the correlation between hsa-miR-375 and ER-α. ACI (August-Copenhagen-Irish) rats were treated with estradiol alone, its inhibitor ellagic acid alone, and estradiol + ellagic acid. ER-α was found to be regulated—among other miRs—by hsa-miR-375. In estradiol-treated cancers, up-regulation of hsa-miR-375 is common and can be reversed by ellagic acid [12, 36].

Ward et al. combined in vitro data with retrospective clinical results. Hsa-miR-375 was found to be down-regulated in Tamoxifen resistant cells. Its re-expression reverses both Tamoxifen resistance and accompanying epithelial-mesenchymal transition (EMT)-like properties in breast cancer. The levels of metadherin (MTDH), a direct target of hsa-miR-375, were measured in patients and correlated inversely with disease-free survival [31]. Hoppe et al. described the connection between miR profiles, ER-α status, and Tamoxifen resistance in postmenopausal patients. Low levels of hsa-miR-375 predicted worse relapse free time, but as opposed to hsa-miR-10a and hsa-miR-126, this effect was not statistically significant [32]. Lyng et al. conducted a similar study in 152 patients with ER-α-positive tumors. Distant recurrence was the statistical endpoint. Of note, patients in the pilot set were matched according to their clinical features, whereas patients in the two validation sets were randomly selected. None of the miRs in the pilot set overlapped with the miRs in the validation sets, and the only statistically highly significant difference between the group of recurrent and non-recurrent patients was the number of tumor-infiltrated lymph nodes [33]. Jonsdottir correlated miR profiles with known clinicopathological factors in 204 lymph node-negative patients with distant metastasis-free survival. Hsa-miR-375 levels were high in ER-α-positive patients. As opposed to hsa-miR-106, hsa-miR-375 was unable to differentiate between patients with or without distant metastases [34]. Giricz et al. investigated hsa-miR-375 in normal tissue, lobular carcinoma in situ and invasive lobular carcinoma (ILC) in formalin-fixed paraffin-embedded (FFPE) specimens of 31 patients and in vitro. The highest levels of hsa-miR-375 were detected in ILC and LCIS adjacent to ILC. Their gene transfer experiments revealed that induced elevation of this microRNA to levels found in human lobular breast cancer specimens changes the ability to maintain appropriate tissue organization. The authors conclude that high levels of hsa-miR-375 contribute to progression from normal tissue to ILC [35]. Since over 90 % [35] of ILC are ER-α-positive, there seems to be a functional connection between ER-α overexpression and elevated levels of hsa-miR-375 as described by Souza et al. [11].

Thus far, the only study with LC as statistical endpoint was performed by Zhou in a cohort of 68 patients. Only FFPE samples with a tumor burden of at least 70 % were included in the analysis. Hsa-miR-9 was able to differentiate between patients with and without local relapse. Hsa-miR-375 was one of the candidate molecules selected in the pilot phase but was finally unable to predict LC in the patients of the validation set. Nevertheless, the authors found a strong correlation between hsa-miR-375 and ER-α status [22].

Taken together, these studies show that to date, it remains a matter of debate whether hsa-miR-375 is of predictive and/or prognostic relevance for early stage breast cancer. In accordance with Jonsdottir et al., our results also show that high levels of hsa-miR-375 lead to a worse outcome [34]. The effect is particularly pronounced in ER-α-positive patients, which coincides with the mechanistic evidence provided by Souza et al. [11]. Discrepancies between our analysis and literature may be explained by our focus on LC in early stage breast cancer. Different clinical settings result in different statistical endpoints, such as disease-free survival and may therefore lead to diverging results. Additionally, sample-related differences may be important. (1) The current study compares tumor tissues with each other, which means that the difference in the miR levels of relapse versus controls must be large to be detected. Iorio et al., e.g., compare normal and tumor tissues [14]. (2) Some studies were performed after microdissection of the specimen [22, 34], whereas in the present analysis, tumor tissue contains stroma. This strategy is based on daily clinical procedures and therefore feasible in diagnostic routine without additional manipulation of the specimen, which adds to the robustness of our results. (3) Whole tissue sections were used in the present analysis since we believe that a given miR—if clinically relevant as a predictor and/or prognosticator—should be able to differentiate between patients even if the tumor cell content is small.

However, a limitation of our study concept is that the cellular signal is either from stromal or tumor tissue. Moreover, there is no validated endogenous reference gene available for breast cancer [37–39]. According to literature, the standard approach is to use small nuclear or small nucleolar RNAs as controls. The reference gene we used was SNORD-61, a small nucleolar RNA, which was validated by Spervelage for neuro-endocrine tumors of the ileum [38].

Beyond the scope of the current analysis, it is important to further elucidate the oncogenic interference of hsa-miR-375 in cancer pathways. This entails in particular the necessity to define mRNA molecules, which contain potential binding sites for hsa-miR-375. In theory, the result would be a down-regulation of a tumor suppressive protein. Our search in five different databases (TargetScan, miRDB, PITA, DIANA, DIANA Cancer) performed in May 2015 resulted in a large variety of potential target molecules. We chose the results given by TargetScan and PITA for two reasons. First, it is generally recommended to select overlapping results from databases that implement different algorithms [40]. Second, these two databases provide a reasonable number of potential targets. TargetScan (Release 6.2) identified 229 putative mRNA targets based on their conserved complementarities to the seed region of the miR [41]. PITA additionally includes secondary structures and binding energies for prediction [42], which consequently resulted in a smaller number of 116 potential targets (Additional file 3). The comparison of both databases revealed 15 overlaps. Among these, we identified one gene that has been validated experimentally by reporter gene assay in breast cancer cells: RASD1 [11]. It belongs to the Ras superfamily of small G proteins, but it does not share key residues in reactive domains with other Ras family members, such as V-Ki-ras2 Kirsten rat sarcoma viral oncogene homolog (KRAS), transforming protein p21 (HRAS), and neuroblastoma ras viral oncogene homolog (NRAS). As opposed to these structurally related molecules, RASD1 inhibits clonal cell expansion suggesting a tumor suppressive role [43]. Additionally, we used the GeneCoDis software [44] to correlate this predicted target with cancer pathways (Panther classification system). According to this analysis, RASD1 is related to the PI3K-pathway (Additional file 4). The involvement in known cancer pathways and its experimental validation as a direct target of hsa-miR-375 makes RASD1 a promising candidate for further evaluation and underlines the potential biological significance of hsa-miR-375 in early stage breast cancer.

## Conclusions

In summary, we could show that high levels of hsa-miR-375 are associated with a significantly higher probability of local relapse, especially in ER-α-positive patients*.* Hence, hsa-miR-375 is an independent predictor for local treatment outcome after breast conserving therapy. Although our results require validation in larger prospective studies, we suggest hsa-miR-375 as an additional molecular marker for treatment choice in early stage breast cancer.

## Methods

### Patients

Between October 1998 and October 2012, 5093 patients were treated for breast cancer at the Department of Radiation Oncology at the Paracelsus Medical University, Salzburg. For the current study, 147 breast cancer patients were selected. According to the 7th edition of the TNM/AJCC classification system, early stage breast cancer includes I, IIA, and IIB (T2 N1). 145/147 (99 %) patients in the current study are in one of this disease stage. 2/147 (1 %) patients (one relapse patient and her matched control) in the pilot cohort had N2 disease. All patients gave their informed consent for surgery, radiotherapy, and systemic treatment. Patients with local relapse were matched to controls without local relapse. Local relapse was defined as re-appearance of the invasive cancer in the same breast, regardless of whether the invasive tumor re-appeared within the former index quadrant, defined by the tumor bed with a 3-cm margin, or not [45–47]. Patients who showed no radiological and histological evidence of local relapse were defined as locally controlled. The study cohort comprised 46 in-breast relapses (in and out quadrant) and 101 matched controls. The patients in our study were selected based on the following criteria: (1) invasive carcinoma; (2) minimum follow-up of five years; (3) the tumor specimen had to be processed by the Department of Pathology at the Paracelsus Medical University; and (4) sufficient FFPE material. Patients were matched according to the year of diagnosis, the type of surgery (mastectomy or lumpectomy), the type of radiotherapy (whole-breast irradiation with percutaneous or intraoperative boost), age, tumor size, lymph node involvement, grading, histology, hormonal receptor status, her2 status, menopausal status, and Ki67 proliferation index. The study was approved by the local ethics committee in Salzburg (*Ethikkommission für das Bundesland Salzburg 415-EP/73/85-2012*).

### Experimental design

The study was conducted in a pilot and a validation phase. In the pilot phase, primary tumors of 32 patients (16 patients who experienced a local relapse versus 16 matched controls who had no relapse) were screened for the most de-regulated miRs by means of a commercially available microarray (Agilent™ Sure Print). This part of the study was carried out by the Comprehensive Biomarker Center™ in Heidelberg. Candidate miRs were selected according to their fold changes (i.e., the ratio of a miR levels in patients with local relapse to those without), and their statistical significance was estimated with the moderated *t* test (LIMMA).

The validation study in an independent cohort was performed in primary tumor tissue of 115 patients and consisted of two steps. In the first step, the candidate miRs were analyzed in 30 patients with local relapse compared to 85 matched controls without relapse. This step was performed by the Laboratory of Molecular Pathology at the Department of Pathology, Paracelsus Medical University, Salzburg. The expression levels of these candidate miRs were assessed with RT-qPCR. In the second step, these results were cross-validated in 19 patients (5 relapses versus 14 matched controls) randomly selected from the validation cohort of 115 patients. This part of the analysis was done at the Department of Pathology of the Ludwig Maximilian University in Munich on a technically different RT-qPCR platform. The experimental design is summarized in the study flow-chart (Additional file 5).

### Samples

Surgical samples were FFPE and archived in the tissue bank of the Department of Pathology at the Paracelsus Medical University, Salzburg. Apart from routine procedures applied to clinical samples, no additional processing was performed. For the current analysis, the whole tissue sections with a maximum proportion of tumor tissue were selected. The tumor burden in the biopsies ranged from 10 to 90 % (see Table 1).

### Pilot phase: microarray

By means of microarray technology, a panel of 1250 miRs was screened. For this purpose, the FFPE samples of the primary tumors were retrieved from the tissue bank, and seven consecutive sections per patient were prepared (depending on tissue size, the thickness of the sections was 2 to 4 μm). Isolation of total miR and chip-based microarrays (Agilent’s Sure PrintG3 Human miRNA microarrays™) were performed according to standard procedures by the Comprehensive Biomarker Center^TM^, Heidelberg.

### Validation phase: RT-qPCR

#### RNA extraction and reverse transcription

Routinely, we used seven sections with a 10-μm thickness for RNA extraction with the miRNeasy FFPE kit (Qiagen^TM^) according to the manufacturer’s instructions. Each sample was treated with DNase I (included in the kit) to avoid DNA contamination. The RNA was eluted in 20 μl water and stored at −80 °C. For RNA quantification, the Qubit™ RNA Assay Kit (Invitrogen^TM^) was used. One hundred thirty nanogram of RNA were reversely transcribed using miScript II RT Kit (Qiagen^TM^) in a total volume of 20 μl. Each reaction was incubated at 37 °C for 60 min and heat-inactivated at 95 °C for 5 min. The complementary DNA (cDNA) was stored at −20 °C until further use.

#### Real-time PCR

Expression levels of candidate miRs were measured by miScript SYBR Green PCR Kit (Qiagen^TM^). Specific PCR target information is provided in Additional file 6: Table S1. The primer assays for each target miR and SNORD61 (catalog number MS00033705) were purchased from Qiagen^TM^. The reaction set up was 0.1 μl cDNA, 1 μl 10× miScript Universal Primer, 5 μl QuantiTect SYBR Green PCR master mix, and 1 μl Primer miScript Primer assay in a total reaction volume of 10 μl. Following the initial activation step at 95 °C for 15 min, the cycling conditions were 40 times 15 s 94 °C, 30 s at 55 °C, and 30 s at 70 °C. All PCR reactions were performed in technical duplicates.

#### qPCR validation

The PCR data were analyzed with the Rotor-Gene 2.0.2.4 software (Qiagen^TM^) according to the suggestions of MIQE guidelines (*m*inimum *i*nformation for publication of *q*uantitative real-time PCR *e*xperiments) [48]. To test the PCR specificity, every reaction was subjected to melting curve analyses. The amplification efficiency of each primer pair was determined by a qPCR assay using a series of 1:5 dilutions of cDNA from the control samples. Each reaction was performed in triplicates. The slope of the resulting standard curve was used to calculate the PCR efficiency according to the following formula: efficiency = 10^(−1/slope)^.

#### Data analysis

The Ct value of the reference gene was subtracted from the Ct value of a given miR to receive the ∆Ct value. Subsequently, the median of all ∆Ct values was calculated. The median of the control group was subtracted from the median of the relapse group to receive a ∆∆Ct value. The fold change was calculated with the *∆∆*Ct method [49]. The non-parametric Mann-Whitney *U* test was used to compare the ∆Ct values of relapse and control groups. Raw *p* values were corrected for multiple testing according to Bonferroni.

### External validation

This second validation step on a technically different platform was performed with the sole purpose of verifying the results of the first validation step; therefore, only hsa-miR-375 was analyzed in this cohort of 19 randomly selected patients. SNORD61 was used as the reference gene.

#### SNORD61 expression analysis

Reverse transcription of 100 ng total RNA was carried out using the RevertAid First Strand cDNA Synthesis Kit (Thermo Scientific^TM^), according to the instructions of the manufacturer. RT-qPCR analyses were performed on a LightCycler 480 instrument using the TaqMan Gene Expression Assay (Life Technologies^TM^) following the instructions of the manual. Briefly, the final reaction mix contained 2 ng cDNA, 1× TaqMan Gene Expression Assay, and 1× TaqMan Gene Expression Mastermix II, no UNG. Single qPCRs analyses were run in duplicates.

#### Hsa-miR-375 expression analysis

Reverse transcription reactions were performed using the TaqMan™ Reverse Transcription Kit (Life Technologies^TM^) according to the manufacturer’s instructions. For single RT reactions, miR-specific RT primers were used and miRs were converted into cDNA in one reaction using 10 ng total RNA. cDNA templates were analyzed applying the TaqMan Small RNA Assay for hsa-miR-375, following the instructions of the manufacturer. Reaction Mixes contained 1× TaqMan Gene Expression Mastermix II, no UNG, 1× TaqMan miRNA assay, and a 1:15 dilution of the RT product. The temperature profile was as follows: 10 min at 95 °C, followed by 40 cycles of 95 °C for 15 s, and 60 °C for 1 min. All samples were analyzed in duplicate reactions. The relative expression of the miRs was calculated by the ΔΔCt comparative threshold method [49].

### Statistics

In the pilot phase, differentially expressed miRs were selected as candidates for further validation according to their fold change and its statistical significance estimated with the moderated *t* test (LIMMA) [50]. Raw *p* values were adjusted for multiple testing according to Benjamini-Hochberg [51]. In order to avoid losing any biomarker with possibly discriminative potential, this rather weak correction method was applied on purpose in the pilot phase. We tried to detect possible clusters in rows (miRs) and columns (samples) of the normalized expression matrix by hierarchical clustering (bottom-up complete linkage clustering using the Euclidean distance as a measure). The dendrogram on top of the expression matrix demonstrates samples which cluster together. Thereby, a miR pattern could be defined that differentiates patients with and without local relapse.

In the validation phase, the Mann-Whitney *U* test was used to compare ∆Ct values, patient-, tumor-, and treatment-related characteristics between groups for statistically significant differences. LC was estimated with the Kaplan-Meier method. The comparison of time-to-event analyses in dependence of differentially expressed miR levels was performed with the log-rank test. Local relapse was defined as an event (= in-breast recurrence). Multivariate analyses were performed with the forward stepwise regression (Cox regression). A raw *p* value <0.05 was considered significant. To adjust for multiple testing, the rather strict Bonferroni correction was applied in this phase of the study in order to test potential markers for statistical robustness [52].

## Additional files

Additional file 1: Figure S1. Comparison of hsa-miR-375 levels in the pilot study. The most prominent single miR that could differentiate relapse from control patients was hsa-miR-375 (LIMMA, raw *p* value 0.009). The box plot shows relapse versus control group, and the expression values of hsa-miR-375 are shown on the *y*-axis. (PDF 24.3 kb) Additional file 2: Table S2. Comparison of subgroups according to receptor status. This table shows that in ER*-*α+ patients, the levels of hsa-miR-375 are higher than in TNBC and ERα− patients, with fold changes of 1.87 and 4.17. The *p* value was estimated with the non-parametric Mann-Whitney *U* test. None of the fold changes is significant. The group of her2-negative patients has slightly higher levels of hsa-miR-375 than the her2-positive patient group, again the difference is not significant. (PDF 124 kb) Additional file 3: Figure S3. Target prediction for hsa-miR-375. The Venn diagram shows the number of predicted targets from TargetScan (blue) http://www.targetscan.org/and PITA (red) http://genie.weizmann.ac.il/pubs/mir07/mir07_data.html; 15 genes were suggested by both algorithms. (PDF 44.5 kb) Additional file 4: Figure S4. Predicted targets of hsa-miR-375 and cancer pathways. Gene enrichment analysis of the predicted targets by GeneCoDis3 software (http://genecodis.cnb.csic.es/). RASD1 was assigned to the PI3K-pathway (http://www.pantherdb.org/pathway/). The numbers on the *x*-axis indicate how many of the 15 “overlapping” genes in Additional file 3: Figure S3 are found in a specific pathway. (PDF 20.2 kb) Additional file 5: Figure S1. Study flow-chart. This additional file shows the various steps of the study. (PDF 42.8 kb) Additional file 6: Table S1. RT-qPCR target information. This table provides information on the exact sequences of the primers that were used for RT-qPCR including the Qiagen™ catalog number. (PDF 29.9 kb)

## Abbreviations

ACI-rats: August-Copenhagen-Irish rats
BCT: breast conserving therapy
EBCTG: Early Breast Cancer Trialist Group
EMT: epithelial-mesenchymal transition
ER-α: estrogen receptor α
FFPE: formalin fixed paraffin embedded
HRAS: transforming protein p21
KRAS: V-Ki-ras2 Kirsten rat sarcoma viral oncogene homolog
LC: local control
LIMMA: linear models for microarray analysis (moderated *t* test)
MIQE: minimum information for publication of quantitative real-time PCR experiments
miR (miRNA): microRNA
MTDH: metadherin
NRAS: neuroblastoma ras viral oncogene homolog
RASD1: dexamethasone-induced ras-related protein 1
RT-qPCR: reverse transcription quantitative polymerase chain reaction
TGF-β: transforming growth factor
