# Supplementary material for: Hsa-miR-375 is a predictor of local control in early stage breast cancer
Source: Clin Epigenetics. 2016 Mar 8;8:28. doi: 10.1186/s13148-016-0198-1 (PMC4784328; doi:10.1186/s13148-016-0198-1)

Figure S2. Comparison of hsa-miR-375 levels in the pilot study:

The most prominent single miR that could differentiate relapse from control patients was hsa-miR-375 (LIMMA, raw p-value 0.009). The box plot shows relapse versus control group, the expression values of hsa-miR-375 are shown on the y-axis.

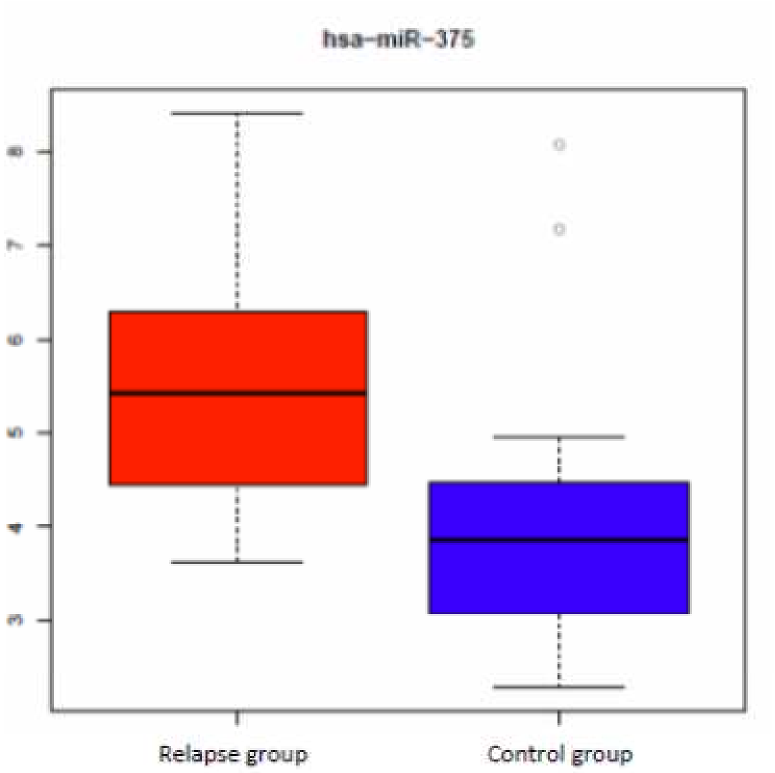

Supplement: Additional file 1: Figure S1. — Comparison of hsa-miR-375 levels in the pilot study. The most prominent single miR that could differentiate relapse from control patients was hsa-miR-375 (LIMMA, raw p value 0.009). The box plot shows relapse versus control group, and the expression values of hsa-miR-375 are shown on the y-axis. (PDF 24.3 kb) [file 13148_2016_198_MOESM1_ESM.pdf]
