# Supplementary material for: Hsa-miR-375 is a predictor of local control in early stage breast cancer
Source: Clin Epigenetics. 2016 Mar 8;8:28. doi: 10.1186/s13148-016-0198-1 (PMC4784328; doi:10.1186/s13148-016-0198-1)
Supplement: Additional file 2: Table S2. — Comparison of subgroups according to receptor status. This table shows that in ER-α+ patients, the levels of hsa-miR-375 are higher than in TNBC and ERα− patients, with fold changes of 1.87 and 4.17. The p value was estimated with the non-parametric Mann-Whitney U test. None of the fold changes is significant. The group of her2-negative patients has slightly higher levels of hsa-miR-375 than the her2-positive patient group, again the difference is not significant. (PDF 124 kb) [file 13148_2016_198_MOESM2_ESM.pdf]

Table S2. Comparison of subgroups according to receptor-status:

This table shows that in ERα+ patients the levels of hsa-miR-375 are higher than in TNBC and ERα- patients, with fold changes of 1.87 and 4.17. The p-value was estimated with the non-parametric Mann-Whitney-U test. None of the fold changes is significant. The group of her2 negative patients has slightly higher levels of hsa-miR-375 than the her2-positive patient group, again the difference is not significant.

<table>
<tr><th></th><th>No. of patients</th><th>Median ΔCt-value</th><th>Fold change</th><th>p-value</th></tr>
<tr><td>ERα+</td><td>90</td><td>4,41</td><td rowspan="2">1,87</td><td rowspan="2">0,53</td></tr>
<tr><td>ERα-</td><td>25</td><td>5,31</td></tr>
<tr><td colspan="5"></td></tr>
<tr><td>ERα+</td><td>90</td><td>4,41</td><td rowspan="2">4,17</td><td rowspan="2">0,24</td></tr>
<tr><td>TNBC*</td><td>11</td><td>6,47</td></tr>
<tr><td colspan="5"></td></tr>
<tr><td>her2-</td><td>62</td><td>4,08</td><td rowspan="2">1,52</td><td rowspan="2">0,66</td></tr>
<tr><td>her2+</td><td>35</td><td>4,68</td></tr>
</table>

*TNBC: triple negative breast cancer
