# Supplementary material for: Hsa-miR-375 is a predictor of local control in early stage breast cancer
Source: Clin Epigenetics. 2016 Mar 8;8:28. doi: 10.1186/s13148-016-0198-1 (PMC4784328; doi:10.1186/s13148-016-0198-1)

Figure S3. Target prediction for hsa-miR-375:

The Venn diagram shows the number of predicted targets from TargetScan (blue) http://www.targetscan.org/ and PITA (red) http://genie.weizmann.ac.il/pubs/mir07/mir07_data.html; 15 genes were suggested by both algorithms.

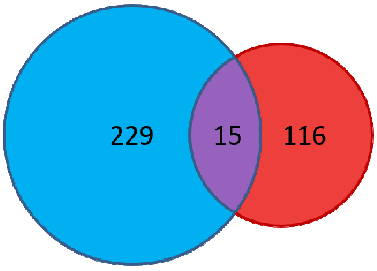

Supplement: Additional file 3: Figure S3. — Target prediction for hsa-miR-375. The Venn diagram shows the number of predicted targets from TargetScan (blue) http://www.targetscan.org/and PITA (red) http://genie.weizmann.ac.il/pubs/mir07/mir07_data.html; 15 genes were suggested by both algorithms. (PDF 44.5 kb) [file 13148_2016_198_MOESM3_ESM.pdf]
