# Supplementary material for: Hsa-miR-375 is a predictor of local control in early stage breast cancer
Source: Clin Epigenetics. 2016 Mar 8;8:28. doi: 10.1186/s13148-016-0198-1 (PMC4784328; doi:10.1186/s13148-016-0198-1)

Figure S4. Predicted targets of hsa-miR-375 and cancer pathways:

Gene enrichment analysis of the predicted targets by GeneCoDis3 software (http://genecodis.cnb.csic.es/). RASD1 was assigned to the PI3K-pathway (http://www.pantherdb.org/pathway/). The numbers on the x-axis indicate how many of the 15 “overlapping” genes in fig. S3 are found in a specific pathway.

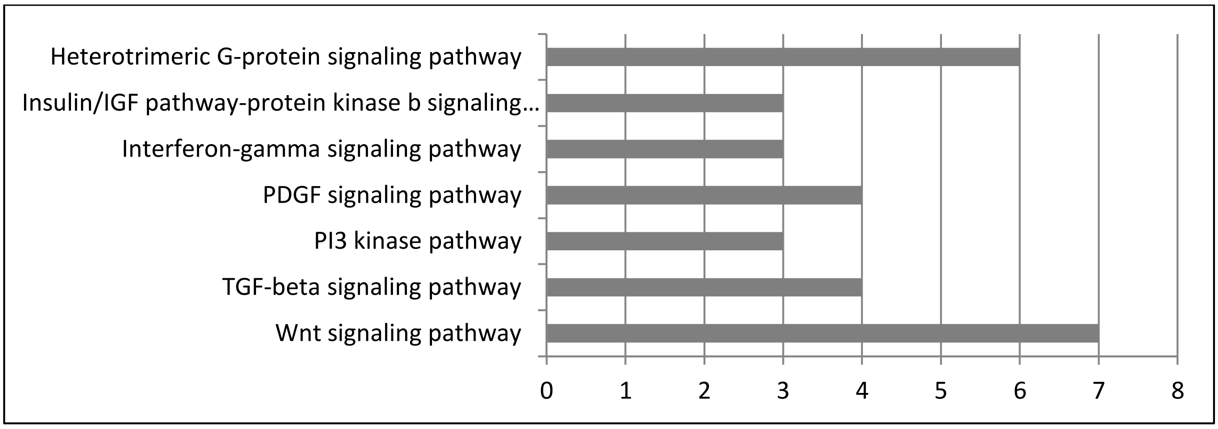

Supplement: Additional file 4: Figure S4. — Predicted targets of hsa-miR-375 and cancer pathways. Gene enrichment analysis of the predicted targets by GeneCoDis3 software (http://genecodis.cnb.csic.es/). RASD1 was assigned to the PI3K-pathway (http://www.pantherdb.org/pathway/). The numbers on the x-axis indicate how many of the 15 “overlapping” genes in Additional file 3: Figure S3 are found in a specific pathway. (PDF 20.2 kb) [file 13148_2016_198_MOESM4_ESM.pdf]
