# Supplementary figures and images for: Hsa-miR-375 is a predictor of local control in early stage breast cancer
Source: Clin Epigenetics. 2016 Mar 8;8:28. doi: 10.1186/s13148-016-0198-1 (PMC4784328; doi:10.1186/s13148-016-0198-1)

Figure S1. Study flow-chart:

This additional file shows the various steps of the study.

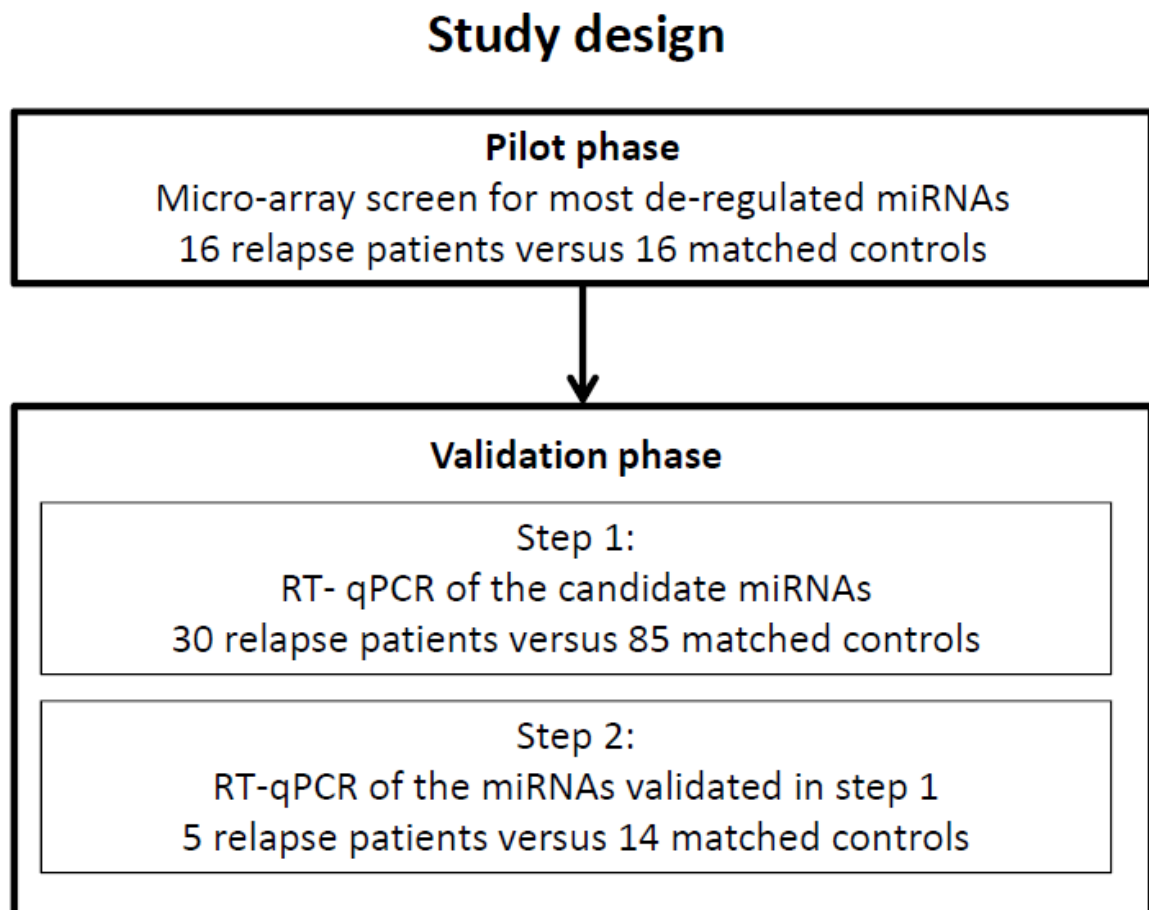

Supplement: Additional file 5: Figure S1. — Study flow-chart. This additional file shows the various steps of the study. (PDF 42.8 kb) [file 13148_2016_198_MOESM5_ESM.pdf]
