# Supplementary material for: Hsa-miR-375 is a predictor of local control in early stage breast cancer
Source: Clin Epigenetics. 2016 Mar 8;8:28. doi: 10.1186/s13148-016-0198-1 (PMC4784328; doi:10.1186/s13148-016-0198-1)
Supplement: Additional file 6: Table S1. — RT-qPCR target information. This table provides information on the exact sequences of the primers that were used for RT-qPCR including the Qiagen™ catalog number. (PDF 29.9 kb) [file 13148_2016_198_MOESM6_ESM.pdf]

Table S1. RT-qPCR target information:

This table provides information on the exact sequences of the primers that were used for RT-qPCR including the Qiagen™ catalogue number.

Mature miRNA	Gene Symbol	Sequence Accession Number	Sequence	Catalogue no.
hsa-miR-125a-5p	HGNC:MIR125a	MIMAT0000443	UCCCUGAGACCCUUUAACCUGUGA	MS00008554
hsa-miR-210-5p	HGNC:MIR210	MIMAT0026475	AGCCCCUGCCCACCGCACACUG	MS00003801
hsa-miR-223-5p	HGNC:MIR223	MIMAT0004570	CGUGUAUUUGACAAGCUGAGUU	MS00003871
hsa-miR-362-3p	HGNC:MIR362	MIMAT0004683	AACACACCUAUUCAAGGAUUCA	MS00009562
hsa-miR-375	HGNC:MIR375	MIMAT0000728	UUUGUUCGUUCGGCUCGCGUGA	MS00031829
hsa-miR-487b-5p	HGNC:MIR487B	MIMAT0026614	GUGGUUAUCCCUGUCCUGUUCG	MS00004298
hsa-miR-532-3p	HGNC:MIR532	MIMAT0004780	CCUCCCACACCCAAGGCUUGCA	MS00004571
hsa-miR-660-5p	HGNC:MIR660	MIMAT0003338	UACCCAUUGCAUAUCGGAGUUG	MS00005383
